# Fistula in Ano

**Published:** 1889-07

**Authors:** C. C. Howard


### FISTULA IN ANO.

[I. H. A., Bureau of Clinical Medicine.]

Mr. President, Ladies and Gentlemen:—The following case, which came to my notice September 10th, 1886, illustrates so well the curative action of a similar drug in a high potency and a single dose, it also so well demonstrates the restriction which should always be put upon any surgical interferences in these cases that I deem it of sufficient interest to report it to you.

Mr. S., æt. thirty-five, a machinist by trade, acknowledging to intemperate habits, with a previous history of gonorrhœa and chancroid but no evidence of syphilis, presented himself at my office on the above date, complaining of soreness, itching, smarting, and burning about the anus. These symptoms were $<$ by scratching, washing, undressing, damp weather, warmth of the bed, and heat in general; and $>$ by cold clear weather.

He said there was a sensation at times as if a hot coal were placed upon the vertex, and that he had an occasional sudden sharp pain, like an electric shock, commencing over the left orbit and extending to the occipital protuberance, lasting about a minute; these pains had become more frequent of late, particularly at any atmospherical change.

He also had a pain extending from the left shoulder to the left testicle with the sensation as if the testicle was being squeezed in a vise; this pain was always $<$ in bed.

There was considerable perspiration about the head, particularly the forehead, and vertex. There was also a profuse perspiration on the genital organs of a sour smell.

Upon examination, I found a complete fistula in ano, the probe entering about half an inch to right of the anus, and entering the rectum just below the internal sphincter. There was a constant slight discharge of bloody pus.

Around the anus and extending to the buttocks there were a large number of papules, bleeding quite profusely upon being scratched; these papules were to be found upon the legs and scattered over the body.

I gave him one powder of Mer. sol.$^{cm}$ and six powders of placebo, one to be taken every second morning, requesting him to call at the end of two weeks, at which time the neuralgic pains had nearly ceased. I saw him at frequent intervals for about three months, when the fistula had entirely healed.

C. C. Howard, M. D.

